# Viscoelastic Properties of Unentangled Multicyclic Polystyrenes

**DOI:** 10.3390/polym10090973

**Published:** 2018-09-01

**Authors:** Zhi-Chao Yan, Md. D. Hossain, Michael J. Monteiro, Dimitris Vlassopoulos

**Affiliations:** 1 Institute of Electronic Structure & Laser, Foundation for Research & Technology Hellas (FORTH), 70013 Heraklion, Greece; yanzhichao09@iccas.ac.cn or yanzhch@szu.edu.cn; 2 College of Materials Science and Engineering, Shenzhen Key Laboratory of Polymer Science and Technology, Guangdong Research Center for Interfacial Engineering of Functional Materials, Nanshan District Key Lab for Biopolymers and Safety Evaluation, Shenzhen University, Shenzhen 518060, China; 3 Australian Institute for Bioengineering and Nanotechnology, The University of Queensland, Brisbane, QLD 4072, Australia; md.hussain@uq.edu.au (M.D.H.); m.monteiro@uq.edu.au (M.J.M.); 4 School of Chemical and Molecular Biosciences, The University of Queensland, Brisbane, QLD 4072, Australia; 5 Department of Materials Science & Technology, University of Crete, 70013 Heraklion, Greece

**Keywords:** multicyclic polymer, hierarchical relaxation, rheology

## Abstract

We report on the viscoelastic properties of linear, monocyclic, and multicyclic polystyrenes with the same low molecular weight. All polymers investigated were found to exhibit unentangled dynamics. For monocyclic polymers without inner loops, a cyclic-Rouse model complemented by the contribution of unlinked chains (whose fraction was determined experimentally) captured the observed rheological response. On the other hand, multicyclic polymers with inner loops were shown to follow a hierarchical cyclic-Rouse relaxation with the outer loops relaxing first, followed by the inner loop relaxation. The influence of unlinked linear chains was less significant in multicyclic polymers with inner loops. The isofrictional zero-shear viscosity decreased with increasing number of constrained segments on the coupling sites, which was attributed to the decreasing loop size and the dilution effect due to the hierarchical relaxation.

## 1. Introduction

Understanding the dynamics of cyclic polymers is a formidable challenge with significant implications in diverse disciplines. For example, in biology, cyclic DNA can form compact conformations in constrained environments [1,2] and become knotted when using appropriate enzymes [3], whereas cyclic RNA has been found to improve the accuracy of genetic transcription [4] and cyclic peptides may exhibit improved stability and bioactivity in comparison with their linear counterparts [5,6]. In materials science, cyclic polymers can be used as templates for the assembly of nanoparticles [7], building blocks for chemical gels [8] and surface modifiers to prevent nanoparticle aggregation [9,10]. In polymer physics, the absence of chain ends makes cyclic polymers unique in terms of structural (e.g., conformation, crystallization) and dynamic (both segmental and global) properties [11,12,13,14,15]. At the same time, the dynamics of entangled cyclic polymers are greatly influenced by the presence of tiny amounts of unlinked chains (often called contaminants [16,17]). In addition, cyclic polymers can serve as probes to detect the dynamics of linear chains since the threaded rings exhibit similar dynamic behavior to their linear blend partner [18,19,20]. Furthermore, cyclic polymers can be thought of as viscosity modifiers since their addition in small amounts to linear matrices increases the viscosity of the latter [21,22,23,24]. On the other hand, there are situations like, for example, the preparation of polysiloxane from cyclic monomers [25], where cyclic polymers are the undesirable byproducts which are difficult to remove, so the evaluation of the effect of cyclic polymers on linear polymer products is a real challenge.

The static and dynamic properties of monocyclic polymers have been widely investigated [11,13,14,15,16,26,27,28,29,30,31,32,33,34,35,36,37]. Due to lack of chain ends, cyclic polymers have a more compact, non-Gaussian conformation with the mean radius of gyration <*R*_g_^2^> proportional to molecular weight ranging from *M*^4/5^ to *M*^2/3^ [11,13], weaker than the scaling in linear chains where <*R*_g_^2^> ~ *M*. Such self-linking structure also results in different stress relaxation mechanism compared to linear chains. In particular, for appropriately purified rings (via liquid chromatography at the critical condition) in order to remove unlinked contaminants [16,32,38], it has been reported that for molecular weights *M* < 5*M*_e_ (*M*_e_ being the entanglement molecular weight), cyclic polymers behave as cyclic-Rouse chains [30,32], with zero-shear viscosity being about half that of the respective linear chains but scaling identically with molecular weight, *η*_0_ ~ *M* [31]. At higher molecular weights, entangled rings relax stress self-similarly, as found experimentally, described for example by the lattice animal or the fractal loopy globule models and confirmed by molecular dynamics simulations [14,16,29,32,39]. The *M*-scaling of the zero-shear viscosity is *η*_0_ ~ *M*^1.4±0.2^, distinctly different from that of linear chains, *η*_0_ ~ *M*^3.4^ [14,31,32]. Similar observations hold for the recoverable compliance of rings, *J*_e_^0^ ~ *M*^1.9±0.2^ for all molecular weights, clearly different from linear chains where *J*_e_^0^ becomes *M*-independent above a critical molecular weight of about 5*M*_e_ [32].

The above progress with monocyclic polymers sets the stage for understanding more complex topologies such as multicyclic polymers which are interesting not only because of the ability to explore the role of well-defined constraints on the properties of a given macromolecule but also because of their implications in decoding the function of biological assemblies [1,2]. The multicyclic topology involves small interconnected local loops and is reminiscent of a structure of a branched polymer, however, when both branches and backbone have no chain ends (see also illustrations in Table 1 below [40,41,42]). Such a structure is expected to relax its stress hierarchically, from outer to inner sections, as established for branched polymers [43,44]. These polymers belong to an emerging class of cyclic-bearing structures including tadpoles [45,46], dumbbells [47], and comb-shaped rings [48]. Understanding their dynamics will not only advance the current state-of-the-art in polymer physics (e.g., exploring the role of constraints on segmental dynamics [42]) but also promote their utility in different applications from composite materials design to biological function. Monteiro and coworkers have synthesized multicyclic polystyrenes with diverse cyclic branches of the same molecular weight [41,49]. They found that the topological constraints due to the presence of cyclic structure increase their glass temperature *T*_g_ compared to linear chains [40]. Molecular dynamic (MD) simulations further showed that the size of multicyclic polymers decreases with the increasing number of loops due to their more compact structure and that their excluded volume in solutions is greater than linear chains [41]. Recently, Pipertzis et al. [42] investigated the segmental dynamics of these multicyclic polymers by dielectric relaxation spectroscopy measurements. The glass temperature was found to increase with the number of constrained segments at coupling sites, indicating the importance of the intramolecular constraints on segmental relaxation. With the link between topology and local dynamics established in multicyclic polymers [42], the next challenge is to explore their global relaxation. In particular, we aim at examining the validity of the hierarchical relaxation mechanism and determining the dependence of the zero-shear viscosity on the polymer structure at isofrictional conditions. In addition, the possible role of residual linear chains contamination needs to be assessed.

In this work, we investigate the viscoelastic properties of monocyclic, multicyclic, and linear polystyrenes with the same total molecular weight, in the unentangled regime. A hierarchical relaxation mechanism accounting for dynamic dilution was employed in order to describe their rheological data. The zero-shear viscosity was adjusted to an isofrictional state and found to decrease with increasing number of constrained segments on the linkers. The effects of linear chain contaminants on the terminal relaxation were determined in monocyclic samples without inner loops by combining experiments and modeling, but not in multicyclic polymers with inner loops. This work establishes the link between multicyclic structures and their linear viscoelastic response.

## 2. Experimental Section

### 2.1. Synthesis and Characterization of Cyclic Structures

The linear, monocyclic, and multicyclic polystyrenes used in this study were synthesized by the copper catalyzed azide−alkyne cycloaddition (CuAAC) [41,49]. The synthesis and detailed characterization of these samples, including impurities and different structures, have been presented in previous publications [41,42,49]. The specific methodology of the multicyclic polystyrene (PS) structures is reported in detail in reference [49]. As there were some subtle changes in the procedure and the additional synthesis of a new multicyclic structure, full details for all the synthesis and characterization of these structures is given in reference [41]. Following the nomenclature in the literature [42,49], we call these samples PSTY and use a code to classify their structure. Their molecular characteristics are listed in Table 1.

Given the complexity of the synthesized structures, some further remarks concerning characterization are in order. The technique of liquid chromatography at the critical condition (LCCC) is considered as the state-of-the-art for purifying ring polymers [16,38]. Whereas this is true, there are situations as explained below, where its use cannot lead to unambiguous results. This technique relies on a separation methodology where the size exclusion and adsorption are matched. This is the case for linear polymers of varying molecular weight. However, the theory suggests that cyclic species have different elution volumes with varying molecular weight. In addition, there are only a few cases where there has been complete baseline resolution between cyclic and linear species, and these are in well-defined systems (i.e., linear l-PSTY and cyclic c-PSTY of identical molecular weight). Moreover, when broad linear and cyclic species are combined (and their characteristic peaks exhibit some overlap), the LCCC chromatograms do not show good separation due to the fact that there is a molecular weight dependence on the cyclic species [38,50,51]. In fact, our HPLC experiments [41] indicate that there is no baseline resolution between cyclic and linear species and the relative proportions of cyclic to linear seem to significantly overestimate what was found by size exclusion chromatography (SEC). This suggests that there is overlap between the cyclic and linear polymer species. The percentage of cyclic was calculated to be only 33%, suggesting that the starting linear polymer adsorbed to the column, possibly due to either the azide or alkyne interactions [41,49]. Hence, there is no evidence that LCCC will work for the CuAAC reaction, as we believe that the azides or alkynes can bind to the column. Therefore, LCCC should be used with caution especially if the chain-ends have an interaction with the column. In addition, the most powerful method to quantify the amount of starting linear remaining is the combination of NMR, FT-IR, MALDI, and SEC [41]. On the other hand, we have shown the accurate use of the log-normal distribution (LND) process to give quantitative data for cyclic purity [52,53]. The LND is now a well-established technique and gives accurate results for our polymers, should the hydrodynamic volume change be already determined [41,42,49].

As mentioned above, a detailed account of the impurities is given in Ref. [41]. Considering sample PSTY-8d, the impurity comes from the coupled linear chains, which amount to about 9% based on LND analysis [41]. Other linear precursors for outer and inner rings were synthesized with the same procedure. In addition, these linear precursors have similar polydispersity to PSTY-8d as shown in Table S1 of Ref. [41]. Hence, we conjecture that all linear precursors have an impurity of 9%. These impurities do affect the multicyclic polymers. The coupled linear byproducts have significant effect on the rheological spectrum, especially the terminal regime since they have double molecular weight. This is further discussed below. The value of coupled linear impurity, 9%, is listed in Table 1 below under the column “*ϕ*_linear_”. The molecular weights of impurities, which correspond to outer and inner loops, are listed under the columns “*M*_l,outer_” and “*M*_l,inner_”, respectively.

### 2.2. Sample Treatment and Rheological Measurements

The samples were molded at 403 K under vacuum in a homemade mold for about 30 min. After cooling to room temperature, the resulting homogeneous specimens were ready to be loaded on the rheometer. Measurements were performed on a strain-controlled rheometer (ARES, TA, New Castle, DE, USA) equipped with a force rebalance transducer (2KFRTN1). Parallel plate geometry was used (homemade stainless steel plates with diameter of 8 mm). The temperature was controlled (±0.1 K) by means of a convection oven. Nitrogen gas flow ensured inert atmosphere in order to reduce the risk of degradation. All measurements were performed at 403 K which allowed for accessing the terminal regime. We focused on dynamic frequency sweeps in the linear regime, which provided information on the frequency-dependent storage and loss moduli (*G*′ and *G*″, respectively). These tests were proceeded by dynamic time sweeps (at fixed frequency and low strain amplitude) and dynamic strain sweeps (at fixed frequency) in order to ensure steady-state conditions (equilibrated samples) and linear viscoelastic (LVE) measurements, respectively. 

## 3. Results and Discussion

**Figure 1:**
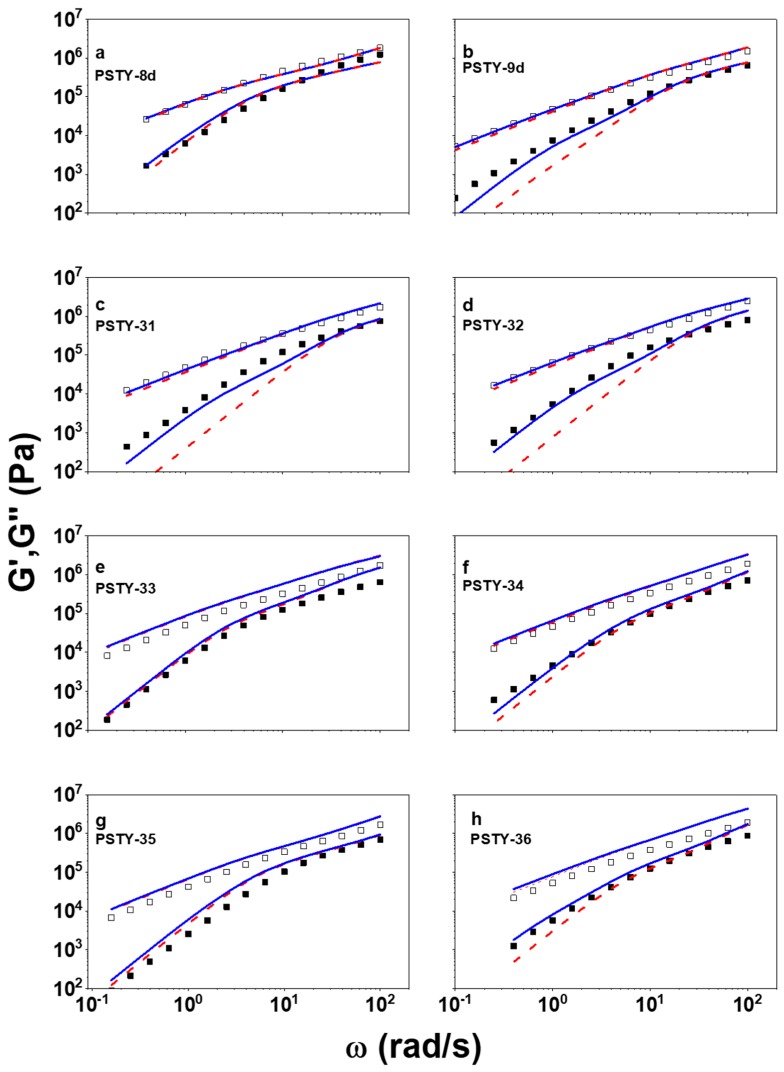
Storage and loss moduli, *G*′ (■) and *G*″ (□), respectively, as functions of oscillatory frequency *ω* for the investigated multicyclic polystyrenes at a reference temperature of 403 K. The blue solid and red dashed curves are model predictions of Equation (6) with and without the contribution of linear contaminants, respectively (see text).

Figure 1 depicts the LVE spectra of the multicyclic polymers investigated, along with the linear precursor and single ring (monocyclic structure), for reference. In the measured temperature, all systems exhibit terminal relaxation in the low-frequency region, preceded by a power-law relaxation at higher frequencies. The power-law exponents deviate from the 1/2 Rouse value and are different for different structures, as will be discussed below. We also note that, unlike the Rouse prediction, the high-frequency moduli do not collapse but *G*″ is slightly higher than *G*′, albeit following the same frequency dependence. This behavior has been observed in other melts and attributed to coupling of high-frequency Rouse and segmental modes [54,55]. In order to analyze the data quantitatively, we divide the multicyclic structure into “outer” and “inner” parts. For example, for sample PSTY-34 (see Table 1 below) the outer parts are the four loops (or rings) and the inner part is the remaining (inner) loop. Based on this structure [41,42,49], the ideal volume fraction of inner ring (*v*_inner_) is 1/3, while the volume fraction of outer rings (*v*_outer_) is 2/3. For samples PSTY-9d, 31, and 32, there are no inner loops. Hence, the total modulus G*_multi_c_ includes contributions of the inner loops and the outer loops and can be written as:

G*_multi_c_ = G*_inner_ + G*_outer_ (1)

Here, we assume that the relaxations of two components (inner and outer) with small molar masses (practically below the entanglement limit) are mutually independent since they follow Rouse dynamics. The ratio *v*_inner_/*v*_outer_ is fixed based on the molecular structure of multicyclic chains, as discussed above (see also Table 1). Further, the multicyclic polymers relax hierarchically, similarly to branched polymers [43,44] as mentioned above, with the outer loops relaxing first in response to an externally imposed stimulus, followed by the relaxation of the inner loops in a diluted environment.

To calculate G*_outer_, we use the cyclic-Rouse model developed by Watanabe et al. [30]:

(2) $$\begin{array}{l} G^{\prime }(\omega )=G_{N}^{0}\frac{2M_{e}}{M_{\mathrm{outer}}}\sum_{p\geq 1}\frac{\omega^{2}\tau_{p}{}^{2}}{1+\omega^{2}\tau_{p}{}^{2}}\\ G^{\prime \prime }(\omega )=G_{N}^{0}\frac{2M_{e}}{M_{\mathrm{outer}}}\sum_{p\geq 1}\frac{\omega \tau_{p}}{1+\omega^{2}\tau_{p}{}^{2}}\\ \tau_{p}=\frac{\tau_{\mathrm{outer}}}{p^{2}},\;\tau_{\mathrm{outer}}=\frac{\tau_{\mathrm{seg}}{\left(M_{\mathrm{outer}}/M_{0}\right)}^{2}}{4} \end{array}$$

with $G_{N}^{0}$ being the plateau modulus, *p* the mode number, *τ_p_* the *p*th Rouse relaxation time, *τ*_seg_ the segmental relaxation time, and *τ*_outer_ the longest Rouse relaxation time of the outer loop (ring), which is 1/4 of the Rouse relaxation time of the respective linear chain with the same molecular weight *M*_outer_. The segmental relaxation time is calculated by the Vogel–Fulcher–Tammann (VFT) equation:

(3) $$\tau_{\mathrm{seg}}=\tau_{0}\exp\left(\frac{B}{T-T_{0}}\right)$$

with *τ*_0_ being the relaxation time at high temperature limit, *B* the activation parameter, and *T*_0_ the “ideal” glass temperature which is below the conventional glass temperature *T*_g_. The VFT parameters (*τ*_0_, *B*, and *T*_0_) are taken from a recent study of the segmental dynamics of these multicyclic polystyrenes [42], where *τ*_0_ = 1 × 10^−12^ s for all samples, *B* = 1518, 1535, 1564, 1563, 1575, 1613, 1593, and 1592 K, and *T*_0_ = 326.1, 325.8, 326.1, 329.4, 327.5, 327.5, 325.6, and 331.2 K, for PSTY-8d, 9d, 31, 32, 33, 34, 35, and 36, respectively.

The calculation of G*_inner_ follows essentially the same equations, also accounting for the dilution effect of the outer loops which act as solvents, reducing the modulus by a dilution factor [54] of ${\left(1-{\bar{v}}_{\mathrm{outer}}\right)}^{2}$:

(4) $$\begin{array}{l} G^{\prime }(\omega )=G_{N}^{0}{\left(1-{\bar{v}}_{\mathrm{outer}}\right)}^{2}\frac{2M_{e}}{M_{\mathrm{inner}}}\sum_{p\geq 1}\frac{\omega^{2}\tau_{p}{}^{2}}{1+\omega^{2}\tau_{p}{}^{2}}\\ G^{\prime \prime }(\omega )=G_{N}^{0}{\left(1-{\bar{v}}_{\mathrm{outer}}\right)}^{2}\frac{2M_{e}}{M_{\mathrm{inner}}}\sum_{p\geq 1}\frac{\omega \tau_{p}}{1+\omega^{2}\tau_{p}{}^{2}}\\ \tau_{p}=\frac{\tau_{\mathrm{inner}}}{p^{2}},\;\tau_{\mathrm{inner}}=\frac{\tau_{\mathrm{outer}}{\left(M_{\mathrm{inner}}/M_{0}\right)}^{2}}{4} \end{array}$$

where *τ*_inner_ is the longest Rouse relaxation time of the inner loop with molecular weight *M*_inner_. The onset of relaxation of the inner loops is set at time *τ*_outer_, i.e., when all outer loops have relaxed. The average fraction of outer loops in the multicyclic polymers is denoted by ${\bar{v}}_{\mathrm{outer}}$. Since there are some remaining reactants and byproducts (e.g., unlinked chains or imperfect multicyclic structures), the value of ${\bar{v}}_{\mathrm{outer}}$ is not exactly the same as that in the ideal situation where all multicyclic polymers are fully grafted. Instead, the average value ${\bar{v}}_{\mathrm{outer}}$ includes the contributions from fully grafted multicyclic polymers, partially grafted multicyclic polymers, coupled multicyclic polymers and ungrafted rings. The average contribution can be calculated by ${\bar{v}}_{\mathrm{outer}}=\sum \phi_{i}v_{\mathrm{outer},i}$, with *ϕ_i_* being the volume fraction of component *i*, and *v*_outer,*i*_ being the fraction of the outer loops in the same component *i*. Similarly, ${\bar{v}}_{\mathrm{inner}}=\sum \phi_{i}v_{\mathrm{inner},i}$ for inner loops. The values of *ϕ_i_*, *v*_outer,*i*_ and *v*_inner,*i*_ are listed in Table S3 in Ref. [41], which are obtained based on the log-normal distribution (LND) fitting of samples’ molecular weight distribution [52]. Let us take PSTY-34 as an example. For ideal fully grafted PSTY-34, *v*_outer_ = 2/3, *ϕ* = 0.97. For partially grafted byproducts, *v*_outer_ = 1/2, *ϕ* = 0.02. For coupled byproducts, *v*_outer_ = 1/2, *ϕ* = 0.01. This yields ${\bar{v}}_{\mathrm{outer}}$ = 0.66 and ${\bar{v}}_{\mathrm{inner}}$ = 0.34. Since fully grafted multicyclic polymers are the dominant component (confirming the high-quality synthesis [41]), the value of ${\bar{v}}_{\mathrm{outer}}$ only slightly deviates from *v*_outer_. For example, for PSTY-34, *v*_outer_ = 2/3 while ${\bar{v}}_{\mathrm{outer}}$ = 0.66. For other multicyclic polymers, the situation is the same. However, the procedure outlined here is general and applies to the analysis of such structures.

PSTY-8d, as the linear precursor of PSTY-9d, contains 9% coupled linear byproduct even after purification by preparative SEC [41]. Other linear precursors for outer and inner rings were synthesized with the same procedure as PSTY-8d [41]. In addition, these linear precursors have the similar polydispersity with PSTY-8d as shown in Table S1 in Ref. [41]. Hence, we conjecture that all linear precursors have around 9% coupled linear byproduct. These contaminants with double molecular weight are ‘inherited’ in the next cyclization step and left in the final cyclic products. Therefore, the contribution of linear contaminants to the modulus, G*_linear_, needs to be accounted for. The calculation of G*_linear_ follows the Rouse model for linear chains.

(5) $$\begin{array}{l} G_{\mathrm{linear}}^{*}=\sum {\bar{v}}_{x}G_{\mathrm{linear},x}^{*}\\ G^{\prime }(\omega )=G_{N}^{0}\frac{M_{e}}{M_{\mathrm{linear},x}}\sum_{p\geq 1}\frac{\omega^{2}\tau_{p}{}^{2}}{1+\omega^{2}\tau_{p}{}^{2}}\\ G^{\prime \prime }(\omega )=G_{N}^{0}\frac{M_{e}}{M_{\mathrm{linear},x}}\sum_{p\geq 1}\frac{\omega \tau_{p}}{1+\omega^{2}\tau_{p}{}^{2}}\\ \tau_{p}=\frac{\tau_{\mathrm{linear},x}}{p^{2}},\;\tau_{\mathrm{linear},x}=\tau_{\mathrm{seg}}{\left(M_{\mathrm{linear},x}/M_{0}\right)}^{2} \end{array}$$

with *τ*_linear,*x*_ the longest Rouse relaxation time of the linear chain (*p* = 1) with molecular weight *M*_linear,*x*_. Here, the subscript *x* represents outer and inner, respectively. *M*_linear,inner_ = 2*M*_inner_ and *M*_linear,outer_ = 2*M*_outer_. For convenience, we classify pure linear PSTY-8d as outer loop in Table 1.

To account for the high-frequency contribution, an extra term G*_high-freq_ is added. The (dominant) contribution from loss moduli *G*″_high-freq_ is approximated as *Aω*, with *A* being 10,000 Pas at 403 K, appropriately shifted from the reported data at 453 K [56,57]. By incorporating the linear contaminant and high-frequency contributions into Equation (1), the final expression for the total modulus G*_tot_ reads:

G*_tot_ = *ϕ*_multi_c_G*_multi_c_ + *ϕ*_linear_G*_linear_ + G*_high-freq_ (6)

with *ϕ*_multi_c_ and *ϕ*_linear_ = 1 − *ϕ*_multi_c_ being the volume fraction of the multicyclic polymers (including cyclic byproducts) and linear chains, respectively. In all fits we used $G_{N}^{0}$ = 0.17 MPa, shifted from the value at 443 K by density compensation, and *M_e_* = 17,500 g/mol [16].

In Figure 1, two model predictions, with (*ϕ*_multi_c_ = 0.91, *ϕ*_linear_ = 0.09) and without (*ϕ*_multi_c_ = 1, *ϕ*_linear_ = 0) contribution of linear contaminants, are plotted along with data. For the case of *ϕ*_linear_ = 0, the prediction significantly underestimates the terminal storage moduli of samples PSTY-9d, 31, and 32, which do not have inner loops. However, if the linear contamination is considered, the predicted storage moduli are increased and almost perfectly match the experimental data. This supports the existence of linear chains contaminants, even though they are not unambiguously resolved in log-normal distribution (LND) fits [41]. In addition, we note that comparison of Figure 1a,b suggests that the linear viscoelastic spectra of linear PSTY-8d and cyclic PSTY-9d are almost identical. To a first approach this is odd, as even at this molecular weight the linear is slower compared to the ring, as demonstrated with different polystyrenes which have been purified via LCCC [31,32]. However, as shown in Table 1, and discussed above, PSTY-9d contains about 9% linear contaminants with double molecular weight. These linear impurities significantly broaden the terminal regime of PSTY-9d, as illustrated in Figure 1b, which compares modeling predictions with (blue solid curve) and without (red dashed curve) considering them.

For samples with inner loops, i.e., PSTY-33, 34, 35, and 36, both predictions with and without linear chain’s contribution can generate good fits to the experimental data without adjustable parameters, hence the modification from linear contaminants does not significantly change the terminal storage moduli. The reason is that the linear contaminants have comparable terminal relaxation time with the inner-loop-containing multicyclic polymers, which relax stress in a hierarchical way and have an extended terminal relaxation. On the other hand, for the multicyclic polymers without inner loops, their terminal relaxation is much faster than that of linear contaminants, therefore linear contamination can significantly extend the terminal regime. Hence, the procedure outlined here indeed establishes the methodology for treating this type of system.

**Figure 2:**
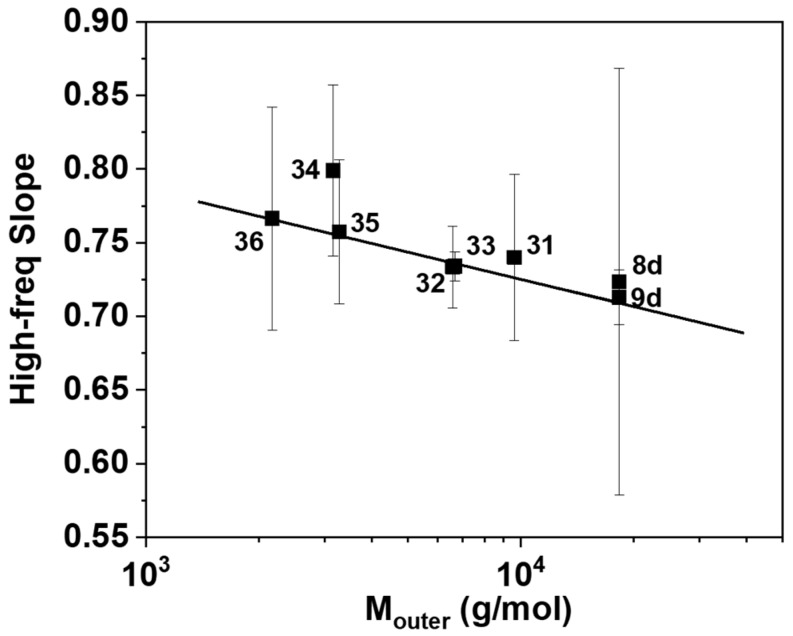
The high-frequency slope as function of outer ring molecular weight *M*_outer_. The solid curve is a linear fit with slope −0.06. Here, the molecular weight of PSTY-8d is treated as *M*_outer_. Error bars reflect standard deviations from each fit.

Next, we examine the high-frequency slopes of *G*′ and *G*″ versus frequency. Their average values range from 0.71 to 0.80 for the different multicyclic structures. This is seen in Figure 2, which depicts them as functions of the molecular weights of the outer loops *M*_outer_ for the different multicyclic structures. Clearly, they are larger than the Rouse prediction (1/2). Such deviation has been widely recognized in linear [54,58,59,60], branched [54,55], and cyclic polymers [32,35]. It has been attributed to the influence of the segmental modes [57,58]. In the present case, we have coupling of the segmental relaxation and the global relaxation of outer loops. The width of the relaxation spectrum for the outer loop is a dominant effect for the high-frequency slope. We argue that the small outer loop exhibits a faster and narrow relaxation process, so that the high-frequency slope is larger.

**Figure 3:**
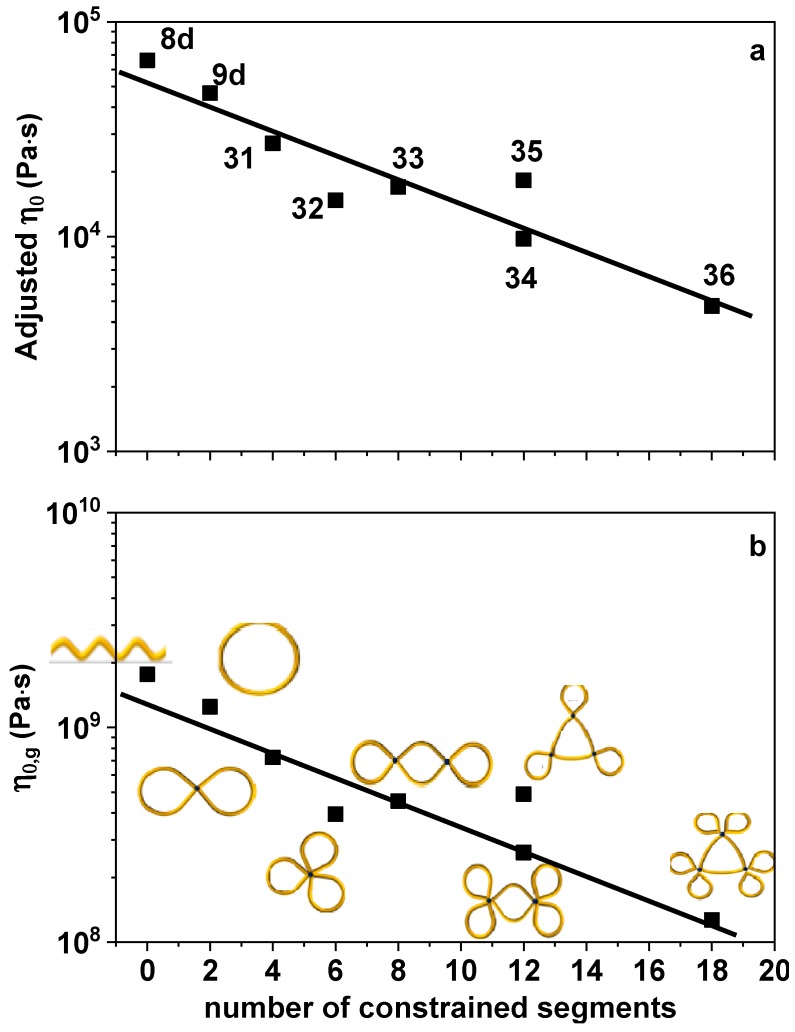
(**a**) The zero-shear viscosity *η*_0_ adjusted to the linear chain’s frictional state, as function of the number of constrained segments of the different multicyclic polystyrenes. The solid curve is the best fit *η*_0_ = *A* × 10^(*B*×*constraint*)^ with *B* = −0.057 and *A* = 5.25 × 10^4^ Pas. (**b**) The zero-shear viscosity *η*_0,g_ adjusted to the *T*_g_ of the respective multicyclic polystyrene as function of the number of constrained segments. The solid curve is the best fit *η*_0_ = *A* × 10^(*B*×*constraint*)^ with *B* = −0.057 and *A* = 1.41 × 10^9^ Pas.

The zero-shear viscosities *η*_0_ are extracted from the LVE data of Figure 1, typically by Carreau-type fitting, η*/*η*_0_ = [1 + (*τ*_0_*ω*)^2^]^(*n*−1)/2^ [61], with η* being the complex viscosity, *ω* being the angular frequency, *η*_0_ being the fitted zero-shear viscosity, and *τ*_0_ and *n* being fit parameters. The thinning exponent was around −0.7. In particular, we found *n* = −0.68, −0.74, −0.70, −0.72, −0.72, −0.78, −0.70, and −0.74 for PSTY-8d, 9d, 31, 32, 33, 34, 35, and 36, respectively. The *η*_0_ values are then adjusted to isofrictional conditions based on the segmental times of Ref. [42]. First, all viscosities are adjusted to the same monomeric frictional state as the linear precursors (PSTY 8d) by multiplying by a factor *τ*_seg,8d_/*τ*_seg,X_, where the subscript X represents the sample code of the multicyclic polymer. Inspired by the analysis of segmental dynamics [42], we plot in Figure 3a the adjusted *η*_0_ as function of the number of intramolecular constrained segments in the vicinity of linkers. The number of intramolecular constrained segments is defined as the number of segments in the vicinity of linkers, which was defined in Ref. [42]. It can be directly read according to the structure of polymers. For example, in PSTY-31 the number of constrained segments is four. A monotonic decrease is observed and represented by an equation of the form η_0_ = *A* × 10^(*B*×*constraint*)^, which provides the best fitting result, where *constraint* represents the number of constrained segments, and *A* and *B* are adjustable parameters with *B* = −0.057 and *A* = 5.25 × 10^4^ Pas. Alternatively, the viscosities at 403 K can be adjusted with respect to their values at respective *T*_g_ of each sample, which is achieved by means of an adjustment factor τ_seg,g_/τ_seg,403 K_, with τ_seg,g_ and τ_seg,403 K_ being the segmental relaxation times at *T*_g_ and 403 K, respectively. We plot the viscosities at *T*_g_, *η*_0,g_, as functions of number of constrained segments in Figure 3b. Since all multicyclic rings have identical total molar mass (Table 1), with increasing constrained segments, the loop size becomes smaller and, due to the dilution effects this results in a decrease of *η*_0,g_. A line in the log-linear plot of the form *η*_0_ = *A* × 10^(*B*×*constraint*)^ with *B* = −0.057 (same as before) and *A* = 1.41 × 10^9^ Pas, represents the best fitting result. The clear scaling of the adjusted viscosity with constraints is encouraging and suggests that it is possible to molecularly design more complex multicyclic structures with tunable rheology.

## 4. Concluding Remarks

The linear viscoelastic properties of unentangled polystyrenes with linear, monocyclic, and multicyclic structures having the same total molecular weight have been investigated. The combination of cyclic-Rouse model and the concept of hierarchical relaxation is found to successfully describe the linear viscoelastic response of these samples. Their modulus includes contributions of the outer and inner loops, whereas the dilution due to the relaxation of the former is accounted for. For samples without inner loops, linear contamination (due to unlinked chains) needs to be considered in order to describe the terminal storage moduli accurately, whereas the contribution of imperfect structures (based on characterization) is minimal. In the high-frequency regime, the power-law dependence of moduli on frequency increases with decreasing *M*_outer_, presumably because the contribution of the early relaxation of branched outer loops. The viscosity at isofrictional state decreases exponentially with the number of constrained segments, which can be explained by the decreasing loop size and the dilution effect. This unique dataset and the associated quantitative analysis can serve as ingredients for the molecular design of composites involving complex multicyclic structures, as well as extending to the more challenging entangled multicyclic polymers.

## Figures and Tables

**Table 1:** Molecular characteristics and dynamic properties of multicyclic polystyrenes at 403 K.

PSTY	Structure	*τ*_seg_ × 10^4^ [s]	*M*_n_ [g/mol]	*M*_outer_ [g/mol]	${\bar{v}}_{\mathrm{outer}}$	*τ*_outer_ [s]	*M*_inner_ [g/mol]	${\bar{v}}_{\mathrm{inner}}$	*τ*_inner_ [s]	*ϕ*_linear_^b^	*M*_l,outer_	*τ*_l,outer_ [s]	*M*_l,inner_ [g/mol]	*τ*_l,inner_ [s]
8d	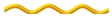	3.74	18,300	18,300 ^a^	1 ^a^	0.25 ^a^	-	-	-	0.09	36,600 ^a^	0.98	-	-
9d	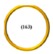	4.32	18,300	18,300	1	0.068	-	-	-	~0.09	36,600	1.1	-	-
31	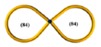	6.80	19,200	9600	1	0.030	-	-	-	~0.09	19,200	0.48	-	-
32	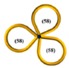	16.7	19,700	6570	1	0.035	-	-	-	~0.09	13,200	0.56	-	-
33	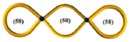	11.5	19,100	6670	0.72	0.025	5750	0.28	0.39	~0.09	13,300	0.39	11,500	0.29
34	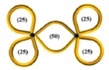	19.0	18,900	3150	0.66	0.0091	6300	0.34	0.17	~0.09	6300	0.14	12,600	0.58
35	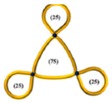	8.68	19,680	3280	0.49	0.0044	9840	0.51	0.21	~0.09	6600	0.072	19,700	0.65
36	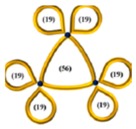	42.6	19,440	2170	0.65	0.0097	6400	0.35	0.19	~0.09	4300	0.15	12,800	1.35

^a^ For convenience, we classify pure PSTY-8d as *outer* loop. ^b^ *ϕ*_linear_ of PSTY-8d was determined by log-normal distribution (LND), while *ϕ*_linear_’s of other samples are approximated as the PSTY-8d value considering their identical synthesis procedure and similar polydispersity.

## References

[1] Witz G., Rechendorff K., Adamcik J., Dietler G. (2011). Conformation of ring polymers in 2D constrained environments. Phys. Rev. Lett..

[2] Halverson J.D., Smrek J., Kremer K., Grosberg A.Y. (2014). From a melt of rings to chromosome territories: The role of topological constraints in genome folding. Rep. Prog. Phys..

[3] Dean F.B., Stasiak A., Koller T., Cozzarelli N.R. (1985). Duplex DNA knots produced by *Escherichia coli* topoisomerase I. Structure and requirements for formation. J. Biol. Chem..

[4] Memczak S., Jens M., Elefsinioti A., Torti F., Krueger J., Rybak A., Maier L., Mackowiak S.D., Gregersen L.H., Munschauer M. (2013). Circular RNAs are a large class of animal RNAs with regulatory potency. Nature.

[5] Craik D.J., Daly N.L., Bond T., Waine C. (1999). Plant cyclotides: A unique family of cyclic and knotted proteins that defines the cyclic cystine knot structural motif. J. Mol. Biol..

[6] Craik D.J. (2006). Seamless proteins tie up their loose ends. Science.

[7] Yu J., Liu Y. (2017). Cyclic polysiloxanes with linked cyclotetrasiloxane subunits. Angew. Chem. Int. Ed..

[8] Zhang K., Lackey M.A., Cui J., Tew G.N. (2011). Gels based on cyclic polymers. J. Am. Chem. Soc..

[9] Morgese G., Trachsel L., Romio M., Divandari M., Ramakrishna S.N., Benetti E.M. (2016). Topological polymer chemistry enters surface science: Linear versus cyclic polymer brushes. Angew. Chem. Int. Ed..

[10] Verbraeken B., Hoogenboom R. (2017). Cyclic polymers: From scientific curiosity to advanced materials for gene delivery and surface modification. Angew. Chem. Int. Ed..

[11] Gooßen S., Brás A.R., Krutyeva M., Sharp M., Falus P., Feoktystov A., Gasser U., Pyckhout-Hintzen W., Wischnewski A., Richter D. (2014). Molecular scale dynamics of large ring polymers. Phys. Rev. Lett..

[12] Ye S., Tang Q., Yang J., Zhang K., Zhao J. (2016). Interfacial diffusion of a single cyclic polymer chain. Soft Matter.

[13] Halverson J.D., Lee W.B., Grest G.S., Grosberg A.Y., Kremer K. (2011). Molecular dynamics simulation study of nonconcatenated ring polymers in a melt. I. Statics. J. Chem. Phys..

[14] Halverson J.D., Lee W.B., Grest G.S., Grosberg A.Y., Kremer K. (2011). Molecular dynamics simulation study of nonconcatenated ring polymers in a melt. II. Dynamics. J. Chem. Phys..

[15] Zardalidis G., Mars J., Allgaier J., Mezger M., Richter D., Floudas G. (2016). Influence of chain topology on polymer crystallization: Poly(ethylene oxide) (PEO) rings vs. linear chains. Soft Matter.

[16] Kapnistos M., Lang M., Vlassopoulos D., Pyckhout-Hintzen W., Richter D., Cho D., Chang T., Rubinstein M. (2008). Unexpected power-law stress relaxation of entangled ring polymers. Nat. Mater..

[17] Halverson J.D., Grest G.S., Grosberg A.Y., Kremer K. (2012). Rheology of ring polymer melts: From linear contaminants to ring-linear blends. Phys. Rev. Lett..

[18] Gooßen S., Krutyeva M., Sharp M., Feoktystov A., Allgaier J., Pyckhout-Hintzen W., Wischnewski A., Richter D. (2015). Sensing polymer chain dynamics through ring topology: A neutron spin echo study. Phys. Rev. Lett..

[19] Kruteva M., Allgaier J., Richter D. (2017). Direct observation of two distinct diffusive modes for polymer rings in linear polymer matrices by pulsed field gradient (PFG) NMR. Macromolecules.

[20] Papadopoulos G.D., Tsalikis D.G., Mavrantzas V.G. (2016). Microscopic dynamics and topology of polymer rings immersed in a host matrix of longer linear polymers: Results from a detailed molecular dynamics simulation study and comparison with experimental data. Polymers.

[21] Vlassopoulos D., Pasquino R., Snijkers F., Tezuka Y. (2014). Progress in the rheology of cyclic polymers. Topological Polymer Chemistry.

[22] Vlassopoulos D. (2016). Macromolecular topology and rheology: Beyond the tube model. Rheol. Acta.

[23] McKenna G.B., Plazek D.J. (1986). The viscosity of blends of linear and cyclic molecules of similar molecular mass. Polym. Commun..

[24] Roovers J. (1988). Viscoelastic properties of polybutadiene rings. Macromolecules.

[25] Endo K., Kobayashi S. (2008). Synthesis and properties of cyclic polymers. New Frontiers in Polymer Synthesis.

[26] Roovers J. (1985). The melt properties of ring polystyrenes. Macromolecules.

[27] Cates M., Deutsch J. (1986). Conjectures on the statistics of ring polymers. J. Phys..

[28] Gooßen S., Brás A.R., Pyckhout-Hintzen W., Wischnewski A., Ricther D., Rubinstein M., Roovers R., Lutz P., Jeong Y., Chang T. (2015). Influence of Solvent Quality on Ring Polymer Dimensions. Macromolecules.

[29] Rubinstein M. (1986). Dynamics of ring polymers in the presence of fixed obstacles. Phys. Rev. Lett..

[30] Watanabe H., Inoue T., Matsumiya Y. (2006). Transient conformational change of bead-spring ring chain during creep process. Macromolecules.

[31] Pasquino R., Vasilakopoulos T.C., Jeong Y.C., Lee H., Rogers S., Sakellariou G., Allgaier J., Takano A., Brás A.R., Chang T. (2013). Viscosity of ring polymer melts. ACS Macro Lett..

[32] Doi Y., Matsubara K., Ohta Y., Nakano T., Kawaguchi D., Takahashi Y., Takano A., Matsushita Y. (2015). Melt rheology of ring polystyrenes with ultrahigh purity. Macromolecules.

[33] Bras A.R., Goossen S., Krutyeva M., Radulescu A., Farago B., Allgaier J., Pyckhout-Hintzen W., Wischnewski A., Richter D. (2014). Compact structure and non-gaussian dynamics of ring polymer melts. Soft Matter.

[34] Tsolou G., Stratikis N., Baig C., Stephanou P.S., Mavrantzas V.G. (2010). Melt structure and dynamics of unentangled polyethylene rings rouse theory, atomistic molecular dynamics simulation, and comparison with the linear analogues. Macromolecules.

[35] Yan Z.C., Costanzo S., Jeong Y., Chang T., Vlassopoulos D. (2016). Linear and nonlinear shear rheology of a marginally entangled ring polymer. Macromolecules.

[36] Grosberg A.Y., Nechaev S.K., Shakhnovich E.I. (1988). The role of topological constraints in the kinetics of collapsed of macromolecules. J. Phys..

[37] Tsalikis D.G., Mavrantzas V.G. (2014). Threading of ring poly(ethylene oxide) molecules by linear chains in the melt. ACS Macro Lett..

[38] Lee H.C., Lee H., Lee W., Chang T., Roovers J. (2000). Fractionation of cyclic polystyrene from linear precursor by HPLC at the chromatographic critical condition. Macromolecules.

[39] Ge T., Panyukoy S., Rubinstein M. (2016). Self-similar conformations and dynamics in entangled melts and solutions of nonconcatenated ring polymers. Macromolecules.

[40] Hossain M.D., Lu D.R., Jia Z.F., Monteiro M.J. (2014). Glass transition temperature of cyclic stars. ACS Macro Lett..

[41] Hossain M.D., Reid J.C., Lu D., Jia Z., Searles D.J., Monteiro M.J. (2018). Influence of constraints within a cyclic polymer on solution properties. Biomacromolecules.

[42] Pipertzis A., Hossain M.D., Monteiro M.J., Floudas G. (2018). Segmental dynamics in multicyclic polystyrenes. Macromolecules.

[43] Kapnistos M., Koutalas G., Hadjichristidis N., Roovers J., Lohse D.J., Vlassopoulos D. (2006). Linear rheology of comb polymers with star-like backbones: Melts and solutions. Rheol. Acta.

[44] Read D.J., Auhl D., Das C., Den Doelder J., Kapnistos M., Vittorias I., McLeish T.C. (2011). Linking models of polymerization and dynamics to predict branched polymer structure and flow. Science.

[45] Doi Y., Ohta Y., Nakamura M., Takano A., Takahashi Y., Matsushita Y. (2013). Precise synthesis and characterization of tadpole-shaped polystyrenes with high purity. Macromolecules.

[46] Doi Y., Takano A., Takahashi Y., Matsushita Y. (2015). Melt rheology of tadpole-shaped polystyrenes. Macromolecules.

[47] Doi Y., Takano A., Matsushita Y. (2016). Synthesis and characterization of dumbbell-shaped polystyrene. Polymer.

[48] Doi Y., Iwasa Y., Watanabe K., Nakamura M., Takano A., Takahashi Y., Matsushita Y. (2016). Synthesis and characterization of comb-shaped ring polystyrenes. Macromolecules.

[49] Hossain M.D., Jia Z.F., Monteiro M.J. (2014). Complex polymer topologies built from tailored multifunctional cyclic polymers. Macromolecules.

[50] Singla S., Zhao T., Beckham H.W. (2003). Purification of cyclic polymers prepared from linear precursors by inclusion complexation of linear byproducts with cyclodextrins. Macromolecules.

[51] Pasch H., Deffieux A., Henze I., Schappacher M., Rique-Lurbet L. (1996). Analysis of macrocyclic polystyrenes. 1. Liquid chromatographic investigations. Macromolecules.

[52] Monteiro M.J. (2015). Fitting molecular weight distributions using a log-normal distribution model. Eur. Polym. J..

[53] Jia Z., Monteiro M.J. (2012). Cyclic polymers: Methods and strategies. J. Polym. Sci. Part A Polym. Chem..

[54] Ruymbeke E.V., Muliawan E.B., Vlassopoulos D., Gao H., Matyjaszewski K. (2011). Melt rheology of star polymers with large number of small arms, prepared by crosslinking poly(*n*-butyl acrylate) macromonomers via ATRP. Eur. Polym. J..

[55] Dorgan J.R., Knauss D.M., Al-Muallum H.A., Huang T., Vlassopoulos D. (2003). Melt Rheology of Dendritically Branched Polystyrenes. Macromolecules.

[56] Inoue T., Matsui H., Osaki K. (1997). Molecular origin of viscoelasticity and chain orientation of glassy polymers. Rheol. Acta.

[57] Inoue T., Okamoto H., Osaki K. (1991). Birefringence of amorphous polymers. 1. Dynamic measurement on polystyrene. Macromolecules.

[58] Ferry J.D. (1980). Viscoelastic Properties of Polymers.

[59] Colby R.H., Fetters L.J., Graessley W.W. (1987). Melt viscosity molecular-weight relationship for linear-polymers. Macromolecules.

[60] Santangelo P.G., Roland C.M. (2001). Interrupted shear flow of unentangled polymer melts. J. Rheol..

[61] Bird R.B., Armstrong R.C., Hassager O. (1987). Dynamics of Polymeric Liquids: Fluid Mechanics.

